# Neuroestrogen-Dependent Transcriptional Activity in the Brains of ERE-Luciferase Reporter Mice following Short- and Long-Term Ovariectomy

**DOI:** 10.1523/ENEURO.0275-19.2019

**Published:** 2019-10-15

**Authors:** Nina E. Baumgartner, Elin M. Grissom, Kevin J. Pollard, Shannon M. McQuillen, Jill M. Daniel

**Affiliations:** 1 Neuroscience Program; 2 Tulane Brain Institute; 3 Department of Psychology, Tulane University, New Orleans, Louisiana 70118

**Keywords:** cortex, estradiol, estrogen, hippocampus, hypothalamus, neuroestrogen

## Abstract

Previous work has demonstrated that estrogen receptors are transcriptionally active in the absence of ovarian estrogens. The current work aims to determine whether brain-derived estrogens influence estrogen receptor-dependent transcription after short- or long-term loss of ovarian function. Experiments were conducted using estrogen response element (ERE)-Luciferase reporter mice, which express the gene for luciferase driven by consensus ERE, allowing for the quantification of ERE-dependent transcription. Brain regions examined were hippocampus, cortex, and hypothalamus. In Experiment 1, short-term (10 d) ovariectomy had no impact on ERE-dependent transcription across brain regions compared with sham surgery. In Experiment 2, chronic intracerebroventricular administration of the aromatase inhibitor letrozole significantly decreased transcriptional activity in 10-d-old ovariectomized mice across brain regions, indicating that the sustained transcription in short-term ovariectomized mice is mediated at least in part via actions of neuroestrogens. Additionally, intracerebroventricular administration of estrogen receptor antagonist ICI-182,780 blocked transcription in 10-d-old ovariectomized mice across brain regions, providing evidence that sustained transcription in ovariectomized mice is estrogen receptor dependent. In Experiment 3, long-term (70 d) ovariectomy significantly decreased ERE-dependent transcription across brain regions, though some residual activity remained. In Experiment 4, chronic intracerebroventricular letrozole administration had no impact on transcription in 70 d ovariectomized mice across brain regions, indicating that the residual ERE-dependent transcription in long-term ovariectomized mice is not mediated by neuroestrogens. Overall, the results indicate that ERE-dependent transcription in the brain continues after ovariectomy and that the actions of neuroestrogens contribute to the maintenance of ERE-dependent transcription in the brain following short-term, but not long-term, loss of ovarian function.

## Visual Abstract

**Figure:**
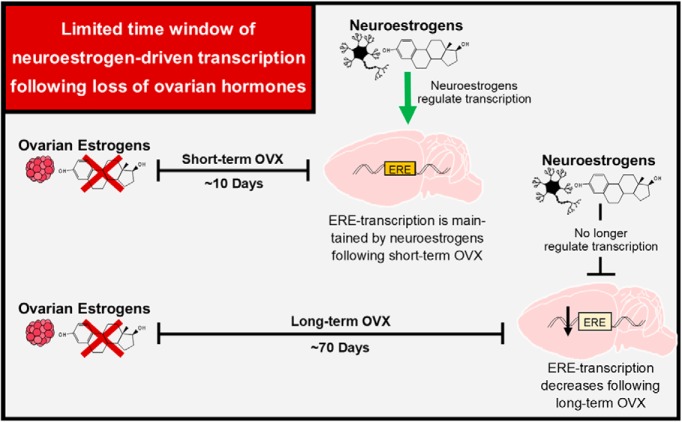

## Significance Statement

Impacts of circulating estrogens on the brain and behavior are widespread and well documented. More recently, the role of neuroestrogens has become a topic of interest. However, the relative contributions of neuroestrogens and ovarian estrogens to estrogen receptor activity in the brain remain unclear, particularly when considering the decline in ovarian estrogens during aging. Previous studies indicate that ovarian estrogens regulate neuroestrogen synthesis in the brain. Therefore, identifying actions of neuroestrogens following the loss of ovarian function is crucial to understanding estrogen receptor function in the brain. Here we demonstrate that estrogen receptors remain transcriptionally active in the brain following long-term loss of ovarian hormones. Neuroestrogens mediate that transcriptional activity for only a limited time following ovariectomy.

## Introduction

Decades of research have shown that estrogens impact cognition by binding to estrogen receptors (ERs) in the brain (Luine, 2014; Daniel et al., 2015; Korol and Pisani, 2015). Nuclear steroid estrogen receptors α and β are expressed throughout the brain, including in the hypothalamus, hippocampus, and cortical areas across species (Shughrue et al., 1997; Mitra et al., 2003; González et al., 2007). Estrogen receptors classically act as transcription factors, binding to estrogen response elements (EREs) to promote transcriptional changes (Hyder et al., 1999). They can also rapidly activate intracellular cascades to impact cellular function on a faster time scale (Luine et al., 2018). Because of the well established impacts of estrogen on learning and memory, much research has focused on the role of estrogen receptors in the hippocampus (Bean et al., 2014). Importantly, hippocampal estrogen receptors have been shown to impact memory (Witty et al., 2012) and act in a transcriptional capacity (Pollard et al., 2018) in the absence of circulating estrogens.

The discovery of brain-derived estrogens has provided a new direction for studying the mechanisms of estrogen receptor activity in the brain. The hippocampus is capable of *de novo* synthesis of estradiol (Prange-Kiel et al., 2003), the main estrogen produced by the ovaries. Other brain regions are known to contain aromatase—the enzyme that converts testosterone to estradiol—including the amygdala, the bed nucleus of stria terminalis, the hypothalamus, and the cerebral cortex (Naftolin et al., 1996; Azcoitia et al., 2011; Stanić et al., 2014; Tabatadze et al., 2014). Locally produced neuroestrogens have been implicated in many aspects of brain function, including hippocampal synaptic plasticity (Kretz et al., 2004), gonadotropin-releasing hormone (GnRH) release from the hypothalamus (Kenealy et al., 2013), and catecholaminergic regulation in the prefrontal cortex (Kokras et al., 2018). Although identical in structure to ovarian estrogens, neuroestrogens appear to act on membrane-bound estrogen receptors to induce rapid changes within cells (Ishii et al., 2007; Wang et al., 2018). It is unknown whether neuroestrogens might also induce genomic actions of nuclear estrogen receptors in the brain, although the rapid actions of membrane estrogen receptors may ultimately lead to transcriptional effects (Lai et al., 2017).

Neuroestrogen synthesis appears to be regulated by circulating estrogens, either produced by the ovaries or given exogenously, via feedback through GnRH neurons in the hypothalamus (Prange-Kiel et al., 2013). In rats, the inhibition of estradiol synthesis in the hippocampus blocks the memory-enhancing effects of systemic estradiol treatment, indicating that the effects of systemic estradiol are mediated through locally produced estradiol (Nelson et al., 2016). In mice, estrogen receptors in the brain remain transcriptionally active after loss of systemic estrogens (Pollard et al., 2018), but it is unclear whether neuroestrogens can activate these receptors in the absence of systemic estrogens. Studies using aromatase inhibitors in recently ovariectomized rodents indicate that neuroestrogens rapidly activate estrogen receptors for at least some period of time following ovariectomy (Tuscher et al., 2016). Ovariectomy in rats reduced brain estradiol levels in the hippocampus, but not in the cortex or amygdala, suggesting a brain region-specific regulation of neuroestrogen synthesis following ovariectomy (Barker and Galea, 2009). Due to the regulatory relationship between circulating estrogens and neuroestrogen synthesis, we hypothesize that the contribution of neuroestrogens to estrogen receptor activation following the loss of ovarian hormones would decrease over time.

The goal of the current project was to test the hypothesis that brain-derived estrogens impact estrogen receptor-dependent transcriptional activity in the brain following short-term, but not long-term, loss of ovarian function. To test this hypothesis, we completed four experiments using the ERE-Luciferase (ERE-Luc) reporter mouse model, which expresses the gene for the firefly enzyme luciferase under the control of consensus ERE sequences (Ciana et al., 2001). In Experiment 1, we measured luciferase activity in the uteri and brains of gonadally intact or short-term (∼10 d) ovariectomized ERE-Luc mice to determine whether ERE-dependent transcription decreases following short-term ovariectomy. In Experiment 2, we administered the aromatase inhibitor letrozole to brains of short-term ovariectomized ERE-Luc mice to determine whether neuroestrogens impact ERE-dependent transcription following short-term ovariectomy. Additionally, we administered the estrogen receptor antagonist ICI 182,780 (ICI) to verify that observed transcription was estrogen receptor dependent. In Experiment 3, we measured luciferase activity in brains of gonadally intact or long-term (∼70 d) ovariectomized ERE-Luc mice to determine whether ERE-dependent transcription decreases following long-term ovariectomy. Finally, in Experiment 4, we administered letrozole to brains of long-term ovariectomized ERE-Luc mice to determine whether neuroestrogens impact ERE-dependent transcription following long-term loss of ovarian hormones.

## Materials and Methods

### Subjects

Adult female heterozygous ERE-Luc model mice (∼70 d of age) were obtained from Charles River Laboratories for Experiments 1 and 2. Adult female heterozygous ERE-Luc model mice (70–110 d of age) were obtained from our onsite breeding colony for Experiments 3 and 4. Animals were group housed in a temperature-controlled AAALAC-accredited vivarium under a 12 h light/dark cycle with *ad libitum* access to phytoestrogen-free food and water. Animal care was performed in accordance with guidelines set by the National Institutes of Health *Guide for the Care and Use of Laboratory Animals* (2011). All animal procedures were performed in accordance with the regulations of the Tulane University animal care committee.

### Ovariectomy surgeries

Mice underwent either ovariectomy (Experiment 1, *n* = 5; Experiment 2, *n* = 12; Experiment 3, *n* = 5; Experiment 4, *n* = 14) or sham surgery (Intact; Experiment 1, *n* = 5; Experiment 2, *n* = 4; Experiment 3, *n* = 5; Experiment 4, *n* = 7) under anesthesia induced by intraperitoneal injection of ketamine (100 mg/kg; Bristol Laboratories) and xylazine (7 mg/kg; MWI Animal Health). Ovariectomy surgeries involved bilateral flank incisions through the skin and muscle wall and the removal of ovaries. Sham surgeries involved bilateral flank incisions through the skin and muscle wall. Incisions were closed using sutures and wound clips. Buprenorphine (0.375 mg/kg; Reckitt Benckiser Health Care) was administered by subcutaneous injection before the start of each surgery. Mice were single housed following surgery. One OVX mouse from Experiment 3 was lost due to complications from anesthesia.

### Estrous cycle tracking

**Figure 1.**
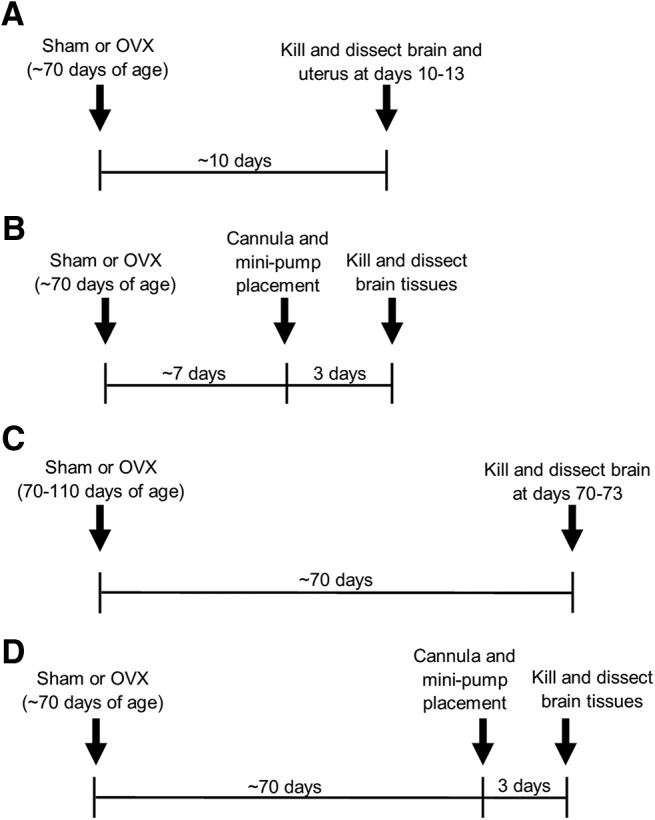
***A–D***, Experimental timelines for Experiment 1 (***A***), Experiment 2 (***B***), Experiment 3 (***C***), and Experiment 4 (***D***). Sham, Sham surgery; OVX, ovariectomy.

The estrous cycles of gonadally intact mice were tracked every day starting 1 d (Experiments 1 and 2) or 55 d (Experiments 3 and 4) after sham or ovariectomy surgery and through the day they were killed using the estrous cycle identification tool described in the study by Byers et al. (2012). Experiments were planned such that the killing of the mice and tissue collection would occur on days that gonadally intact mice were in proestrus with pair-matched ovariectomized controls killed on the same days. See Figure 1*A–D* for summaries of experiment timelines.

#### Experiment 1

Animals were killed on the first day of proestrus that occurred at least 10 d following sham surgery in the gonadally intact animal of each pair.

#### Experiment 2

Animals underwent stereotaxic surgery on the day after the first proestrus day that occurred at least 7 d following sham surgery in the gonadally intact animal of each pair. All mice were killed 3 d after stereotaxic surgery on days that gonadally intact mice were in proestrus.

#### Experiment 3

Animals were killed on the first day of proestrus that occurred at least 70 d following sham surgery in the gonadally intact animal of each pair.

#### Experiment 4

Animals underwent stereotaxic surgery on the day after the first proestrus day that occurred at least 67 d following sham surgery in the gonadally intact animal of each pair. All mice were killed 3 d after stereotaxic surgery on days that gonadally intact mice were in proestrus.

### Cannula and mini-pump implantations

#### Experiment 2

Mice were anesthetized with ketamine and xylazine as described above and administered buprenorphine as an analgesic. Mice were then placed into a stereotaxic frame. An incision was made in the scalp and fascia that overlie the skull. A hole was drilled in the skull, and a cannula (brain infusion kits, Alzet) was lowered through the hole to the appropriate depth to reach the right lateral ventricle (relative to bregma: anteroposterior, −0.5 mm; mediolateral, −1.1 mm; dorsoventral, −2.5 mm) and anchored to the skull with Super Glue and dental acrylic. The cannula was connected to an osmotic mini-pump (flow rate, 0.5 μl/h; Alzet) by vinyl tubing for drug delivery. Gonadally intact animals received vehicle (*n* = 4) containing 10% DMSO (Sigma-Aldrich) in artificial CSF (aCSF; Tocris Bioscience). Ovariectomized animals received vehicle (*n* = 4), the aromatase inhibitor letrozole (0.1 μg/μl; Bachem; *n* = 4), or the estrogen receptor antagonist ICI 182,780 (*n* = 4; 0.3 μg/μl; Sigma-Aldrich). The pump was implanted subcutaneously in the nape of the neck, and the cannula was inserted after the pump began pumping.

#### Experiment 4

Mice received a cannula directed to the right lateral ventricle connected to mini-pumps (flow rate, 0.5 μl/h) in a procedure identical to that described in Experiment 2. Gonadally intact mice received mini-pumps that delivered vehicle (*n* = 7) containing 10% DMSO in aCSF. Ovariectomized mice received mini-pumps containing either vehicle (*n* = 7) or letrozole (0.1 μg/μl; *n* = 7).

### Euthanasia and tissue processing

All animals were anesthetized by intraperitoneal injection of ketamine and xylazine and were killed during the same time window (10:00 A.M. to 12:00 P.M.), which coincided with the timing of daily vaginal smears. The right hippocampus, hypothalamus, right parietal cortex, and uterus (Experiment 1 only) were dissected out and quick frozen on dry ice, and stored at −80°C until processing. In Experiment 1, the hypothalamus for one gonadally intact mouse and one ovariectomized mouse, the hippocampus for one gonadally intact mouse, and the uterus for one gonadally intact mouse and one ovariectomized mouse were damaged and excluded from statistical analysis. In Experiment 2, the hippocampus from one mouse receiving ICI 182,780 was damaged and excluded from statistical analysis. Final samples sizes for all experiments are listed in Tables 1–4. In Experiments 2, 3, and 4, a 1-cm-long segment of the right uterus was dissected out and weighed to confirm ovariectomy status.

**Table 1:** Experiment 1 statistics

Figure	Statistical test	Independent variables	Sample size	Statistics	Observed power	*Post hoc* comparison (if applicable)	*Post hoc* statistics
2*A*	Two-way ANOVA	Brain region (cortex, hypothalamus, hippocampus)Treatment (Intact, OVX)	Cortex:Intact *n* = 5OVX *n* = 5Hypothalamus:Intact *n* = 4OVX *n* = 4Hippocampus:Intact *n* = 4OVX *n* = 5				
		Main effect of brain region		*F*_(2,21)_ = 0.903; *p* = 0.420	0.0185		
		Main effect of treatment		*F*_(1,21)_ = 0.780; *p* = 0.387	0.135		
		Brain region × treatment interaction		*F*_(2,21)_ = 0.903; *p* = 0.420	0.1845		
2*B*	One-way ANOVA	Treatment (Intact, OVX)	Intact *n* = 4OVX *n* = 4	*F*_(1,6)_ = 5.556; *p* = 0.057	0.624		

**Table 2:** Experiment 2 statistics

Figure	Statistical test	Independent variable (s)	Sample size	Statistics	Observed power	*Post hoc* comparison (if applicable)	*Post hoc* statistics
3	Two-way ANOVA	Brain region (cortex, hypothalamus, hippocampus)Treatment (Intact + aCSF, OVX + aCSF, OVX + ICI, OVX + Let)	Cortex:Intact + aCSF *n* = 4OVX + aCSF *n* = 4OVX + ICI *n* = 4OVX + Let *n* = 4Hypothalamus:Intact + aCSF *n* = 4OVX + aCSF *n* = 4OVX + ICI *n* = 4OVX + Let *n* = 4Hippocampus:Intact + aCSF *n* = 4OVX + aCSF *n* = 4OVX + ICI *n* = 3OVX + Let *n* = 4				
		Main effect of brain region		*F*_(2,35)_ = 0.031; *p* = 0.970	0.054		
		Main effect of treatment		*F*_(3,35)_ = 5.448; *p = 0.004	0.909	Intact + aCSF vs OVX + aCSF	*p* = 0.527
						OVX + aCSF vs OVX + ICI	*p = 0.002
						OVX + aCSF vs OVX + Let	*p = 0.030
		Brain region × treatment interaction		*F*_(6,35)_ = 1.015; *p* = 0.432	0.345		

Let, Letrozole.

*Significant.

**Table 3:** Experiment 3 statistics

Figure	Statistical test	Independent variable (s)	Sample size	Statistics	Observed power	*Post hoc* comparison (if applicable)	*Post hoc* statistics
4	Two-way ANOVA	Brain region (cortex, hypothalamus, hippocampus)Treatment (Intact, OVX)	Cortex:Intact *n* = 5OVX *n* = 4Hypothalamus:Intact *n* = 5 OVX *n* = 4 Hippocampus: Intact *n* = 5 OVX *n* = 4				
		Main effect of brain region		*F*_(2,21)_ = 0.486; *p* = 0.622	0.119		
		Main effect of treatment		*F*_(1,21)_ = 13.327; *p = 0.001	0.936		
		Brain region × treatment interaction		*F*_(2,21)_ = 0.486; *p* = 0.622	0.119		

*Significant.

**Table 4:** Experiment 4 statistics

Figure	Statistical test	Independent variable (s)	Sample size	Statistics	Observed power	*Post hoc* comparison (if applicable)	*Post hoc* statistics
5	Two-way ANOVA	Brain region (cortex, hypothalamus, hippocampus)Treatment (Intact + aCSF, OVX + aCSF, OVX + Let)	Cortex:Intact + aCSF *n* = 7OVX + aCSF *n* = 7OVX + Let *n* = 7Hypothalamus:Intact + aCSF *n* = 7OVX + aCSF *n* = 7OVX + Let *n* = 7Hippocampus:Intact + aCSF *n* = 7OVX + aCSF *n* = 7OVX + Let *n* = 7				
		Main effect of brain region		*F*_(2,54)_ = 0.220; *p* = 0.804	0.083		
		Main effect of treatment		*F*_(2,54)_ = 5.785; *p = 0.005	0.851	Intact + aCSF vs OVX + aCSF	*p = 0.016
						OVX + aCSF vs OVX + Let	*p* = 0.898
		Brain region × treatment interaction		*F*_(4,54)_ = 0.589; *p* = 0.672	0.183		

Let, Letrozole.

*Significant.

Tissue was homogenized in luciferase reporter lysis buffer (Promega) and processed according to manufacturer instructions. Homogenate was flash frozen on dry ice and incubated for 15 min. Samples were then rapidly thawed at 37°C and centrifuged for 30 min at 4900 × *g* at 4°C. Supernatant was collected for luciferase enzyme expression assay and Lowry protein assay.

### Luciferase assays

Luciferase enzyme expression was measured using the Promega Luciferase Assay System. Briefly, 100 μl of luciferase assay substrate was added to 20 μl of sample, and the resulting light intensity generated by the lysate was measured by luminometer (PerkinElmer) in triplicate. This process was then repeated using samples in the reverse order to account for signal decay over the course of luminometer readings. Measurements were averaged to obtain the relative light units (RLU), which were normalized to protein concentrations obtained from Lowry protein assays (BCA Protein Assay Kit, Bio-Rad). The final value generated as a measurement of luciferase content was in RLU per microgram of protein (Ciana et al., 2003; Pollard et al., 2018).

### Statistical analyses

Researchers were blind to experimental group during the luciferase assays and statistical analyses. All statistical analyses were conducted using SPSS software (IBM).

#### Experiment 1

Luciferase content in the brain was analyzed by two-way ANOVA with the factors treatment (Intact, OVX) and brain region (cortex, hypothalamus, hippocampus). Luciferase content in the uterus was analyzed by one-way ANOVA with the factor treatment (Intact, OVX).

#### Experiment 2

Luciferase content in the brain was analyzed by two-way ANOVA with factors treatment (Intact + aCSF, OVX + aCSF, OVX + letrozole, OVX + ICI) and brain region (cortex, hypothalamus, hippocampus). A significant main effect of treatment was probed by the Dunnett’s *post hoc* test, which compares treatments with a single control group (OVX + aCSF).

#### Experiment 3

Luciferase content in the brain was analyzed by two-way ANOVA with the factors treatment (Intact, OVX) and brain region (cortex, hypothalamus, hippocampus).

#### Experiment 4

Luciferase content in the brain was analyzed by two-way ANOVA with the factors treatment (Intact + aCSF, OVX + aCSF, OVX + letrozole) and brain region (cortex, hypothalamus, hippocampus). A significant main effect of treatment was probed by the Dunnett’s *post hoc* test, which compares treatments with a single control group (OVX + aCSF).

## Results

### Experiment 1: impact of short-term ovariectomy on ERE-dependent transcription

The goal of the first experiment was to test the hypothesis that ERE-dependent transcription levels in the brain are maintained for a short time period following ovariectomy.

**Figure 2.**
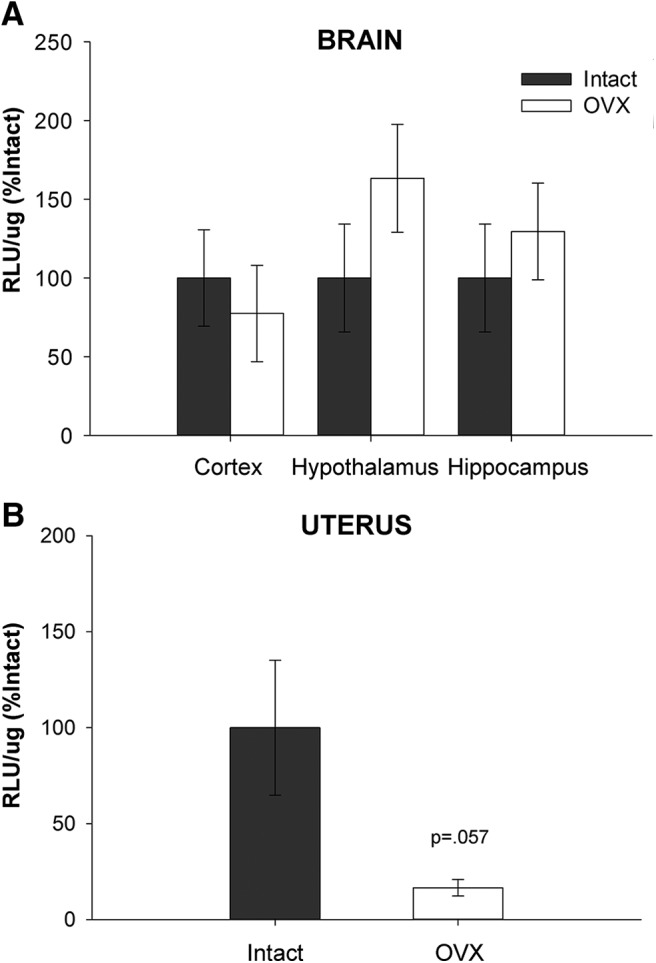
Impact of short-term ovariectomy on ERE-dependent transcription. ERE-Luciferase reporter mice were ovariectomized (OVX) or underwent sham surgery (Intact). Approximately ten days later, mice were killed and luciferase activity was measured in the brain (cortex, hypothalamus, and hippocampus) and uterus. ***A***, Short-term ovariectomy had no impact on luciferase activity as measured by relative light units per microgram of protein (RLU/μg) across brain regions. There was no effect of brain region or interaction between treatment and brain region. ***B***, Short-term ovariectomy resulted in a near significant (*p* = .057) decrease in luciferase activity in the uterus. Data are presented as means ± SEM normalized to percent Intact.

As illustrated in Figure 2*A*, there was no main effect of treatment (*F*_(1,21)_ = 0.780, *p* = 0.387) or brain region (*F*_(2,21)_ = 0.903, *p* = 0.420) on luciferase activity in the brain. As illustrated in Figure 2*B*, there was a nearly significant effect (*F*_(1,6)_ = 5.556, *p* = 0.057) of treatment in the uterus, with ovariectomy decreasing uterine luciferase activity. Results show that ERE-dependent transcription in the brain, but not in the uterus, is maintained following short-term ovariectomy.

### Experiment 2: impact of aromatase inhibition and estrogen receptor antagonism on ERE-dependent transcription following short-term ovariectomy

Results of Experiment 1 indicated that ERE-dependent transcription in the brain is not impacted by short-term ovariectomy. The goal of the second experiment was to test the hypothesis that sustained levels of ERE-dependent transcription in the brain following short-term ovariectomy are due to the actions of neuroestrogens. Additionally, we aimed to confirm the validity of our mouse model by determining whether luciferase activity was blocked by estrogen receptor antagonism.

**Figure 3.**
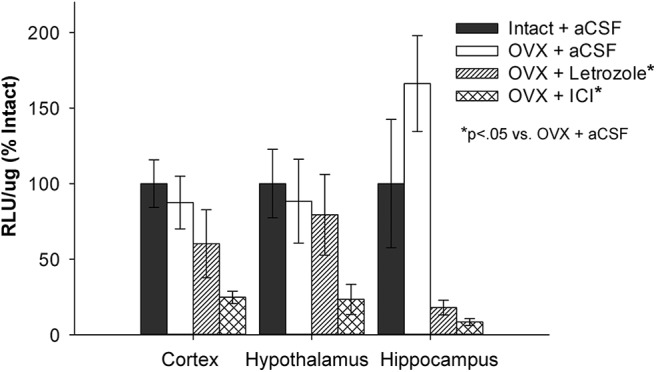
Impact of aromatase inhibition and estrogen receptor antagonism on ERE-dependent transcription following short-term ovariectomy. ERE-Luciferase reporter mice were ovariectomized (OVX) or underwent sham surgery (Intact). Beginning ∼7 days after surgeries, OVX mice received chronic i.c.v. delivery of vehicle (OVX + aCSF), the aromatase inhibitor letrozole (OVX + Letrozole) or the estrogen receptor antagonist ICI 182,780 (OVX + ICI). Intact mice received vehicle (Intact + aCSF). Three days later, mice were killed and luciferase activity was measured in the cortex, hypothalamus, and hippocampus. There was a main effect of treatment (*p* < .05) on luciferase activity as measured by relative light units per microgram of protein (RLU/μg) across brain regions. Post hoc testing revealed that luciferase activity was not impacted by short-term ovariectomy (OVX + aCSF vs. Intact + aCSF), but was decreased in ovariectomized mice by aromatase inhibition (OVX + aCSF vs. OVX + Letrozole, *p* < .05) and by estrogen receptor antagonism (OVX + aCSF vs. OVX + ICI, *p* < .05). There was no effect of brain region or interaction between treatment and brain region. Data are presented means ± SEM normalized to percent Intact + aCSF.

As illustrated in Figure 3, there was a main effect of treatment (*F*_(3,35)_ = 5.448, *p* = 0.004). *Post hoc* analyses revealed that, consistent with the results of Experiment 1, luciferase activity was not impacted by short-term ovariectomy (Intact + aCSF vs OVX + aCSF; *p* = 0.527). Furthermore, aromatase inhibition via the administration of letrozole significantly decreased luciferase activity following short-term ovariectomy (OVX + aCSF vs OVX + letrozole; *p* = 0.030). Antagonism of estrogen receptors via the administration of ICI 182,780 significantly decreased luciferase activity compared with ovariectomized control treatment (OVX + aCSF vs OVX + ICI; *p* = 0.002). There was no significant effect of brain region (*F*_(2,35)_ = 0.031; *p* = 0.970) and no significant interaction (*F*_(6,35)_ = 1.015; *p* = 0.432) between treatment and brain region. Results indicate that following short-term ovariectomy, the maintenance of ERE-dependent transcriptional activity in the brain is driven by neuroestrogens. Furthermore, they demonstrate the validity of the model in that antagonism of estrogen receptors blocks transcriptional activity.

### Experiment 3: impact of long-term ovariectomy on ERE-dependent transcription

Results of the first two experiments indicated that a short-term loss of ovarian hormones has no impact on levels of ERE-dependent transcription in the brain. Therefore, the goal of Experiment 3 was to test the hypothesis that long-term loss of ovarian function will result in a significant decrease in brain levels of ERE-dependent transcription.

**Figure 4.**
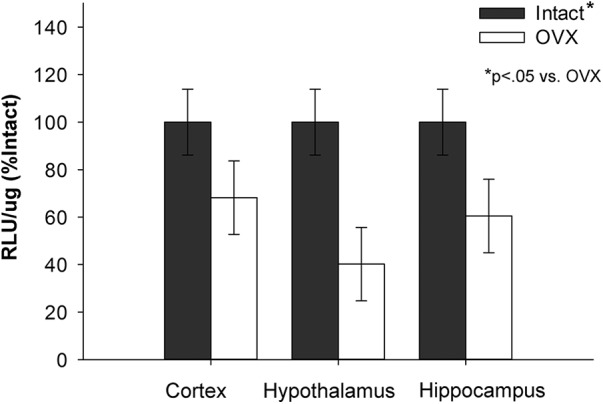
Impact of long-term ovariectomy on ERE-dependent transcription. ERE-Luciferase reporter mice were ovariectomized (OVX) or underwent sham surgery (Intact). Approximately 70 days later, mice were killed and luciferase activity was measured in the cortex, hypothalamus, and hippocampus. Long-term ovariectomy significantly decreased luciferase activity as measured by relative light units per microgram of protein (RLU/μg) across brain regions (*p* < .05). There was no effect of brain region or interaction between treatment and brain region. Data are presented as means ± SEM normalized to percent Intact.

As illustrated in Figure 4, there was a main effect of treatment (*F*_(1,21)_ = 13.327; *p* = 0.001), indicating that long-term ovariectomy significantly decreases luciferase activity in the brain compared with gonadally intact controls. There was no effect of brain region (*F*_(2,21)_ = 0.486; *p* = 0.622) and no interaction (*F*_(2,21)_ = 0.486; *p* = 0.622) between treatment and brain region. Despite the significant decrease in luciferase activity in the brain following long-term ovariectomy, some residual transcriptional activity was evident in all three brain regions 70 d after the loss of ovarian function.

### Experiment 4: impact of aromatase inhibition on ERE-dependent transcription following long-term ovariectomy

Results of Experiment 3 indicate that long-term ovarian hormone deprivation attenuates but does not completely block ERE-dependent transcription in the brain. The goal of the final experiment was to explore the contributions of neuroestrogens to the residual ERE-dependent transcriptional activity evident following long-term loss of ovarian hormones. Because the model was validated as being dependent on estrogen receptors in Experiment 2, the ICI 182,780 treatment group was not included in this final experiment.

**Figure 5.**
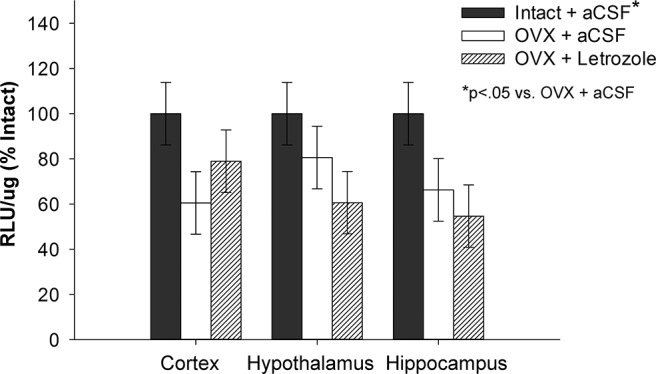
Impact of aromatase inhibition on ERE-dependent transcription following long-term ovariectomy. ERE-Luciferase reporter mice were ovariectomized (OVX) or underwent sham surgery (Intact). Beginning ∼70 days after surgeries, OVX mice received chronic i.c.v. delivery of vehicle (OVX + aCSF), or the aromatase inhibitor letrozole (OVX + Letrozole). Intact mice received vehicle (Intact + aCSF). Three days later, mice were killed and luciferase activity was measured in the cortex, hypothalamus, and hippocampus. There was a main effect of treatment (*p* < .05) on luciferase activity as measured by relative light units per microgram of protein (RLU/μg) across brain regions. Post hoc testing revealed that luciferase activity was significantly decreased following long-term ovariectomy (OVX + aCSF vs. Intact + aCSF, *p* < .05), but was not impacted by aromatase inhibition in ovariectomized mice (OVX + aCSF vs. OVX + Letrozole). There was no effect of brain region or interaction between treatment and brain region. Data are presented as means ± SEM normalized to percent Intact + aCSF.

As illustrated in Figure 5, there was a main effect of treatment (*F*_(2,54)_ = 5.785; *p* = 0.005). *Post hoc* analyses revealed that, consistent with the results of Experiment 3, luciferase activity was decreased following long-term ovariectomy (Intact + aCSF vs OVX + aCSF; *p* = 0.016). Aromatase inhibition via the administration of letrozole did not impact luciferase activity following long-term ovariectomy compared with ovariectomized control treatment (OVX + aCSF vs OVX + letrozole; *p* = 0.898). There was no significant effect of brain region (*F*_(2,54)_ = 0.220; *p* = 0.804) and no significant interaction (*F*_(4,54)_ = 0.589; *p* = 0.672) between treatment and brain region. Results indicate that neuroestrogens do not contribute to the residual ERE-dependent transcription evident following long-term ovariectomy.

## Discussion

Results of the present study indicate that ERE-dependent transcription in the brain continues after the loss of ovarian hormones, and that brain-derived estradiol mediates that transcriptional activity for at least a limited period of time following ovariectomy. Specifically, we showed that ERE-dependent transcription in the cortex, hypothalamus, and hippocampus is sustained at least 10 d after ovariectomy. Seventy days after ovariectomy, ERE-dependent transcription significantly decreases in the brain, although some residual activity remains. Furthermore, we showed that intracerebroventricular administration of the aromatase inhibitor letrozole attenuates ERE-dependent transcription in the brain following short-term, but not long-term, ovariectomy. Collectively, these data reveal that neuroestrogens impact ERE-dependent transcription for only a limited time window after the loss of ovarian hormones.

In Experiments 1 and 2, we showed that there was no decrease in ERE-dependent transcription in any of the brain regions analyzed 10 d after ovariectomy, a time point at which ovarian estrogens are no longer circulating throughout the body (Woolley and McEwen, 1993). We showed a nearly significant decrease in ERE-dependent transcription in the uterus at the same time point—consistent with previous work using the ERE-Luc mouse model (Ciana et al., 2003)—indicating that this maintained transcription is specific to the brain. To understand the mechanism through which transcription in the brain is maintained, we administered the aromatase inhibitor letrozole to the brain to block neuroestrogen synthesis. Blocking neuroestrogen synthesis resulted in a significant decrease in ERE-dependent transcription following short-term ovariectomy. Current results indicate that this decrease occurs across brain areas, as revealed by a statistical main effect of treatment and a lack of an interactive effect of treatment and brain area. However, the effect of letrozole on transcription is numerically larger in the hippocampus than it is in the cortex and hypothalamus. Future experiments with a larger sample size and thus more statistical power could reveal differential effects across brain areas.

The present results are consistent with the current literature on neuroestrogens in short-term ovariectomized animal models, which predominately has been focused on the hippocampus. For example, letrozole administration negatively impacted memory consolidation (Tuscher et al., 2016) and hippocampal spine synaptic density (Zhou et al., 2010) in recently ovariectomized mice and changed serotonergic and catecholaminergic turnover rates in the hippocampus and prefrontal cortex of ovariectomized rats (Kokras et al., 2018). Aromatase activity has been documented in several brain regions, including the hypothalamus and cerebral cortex (Naftolin et al., 1996; Azcoitia et al., 2011; Stanić et al., 2014; Tabatadze et al., 2014). However, the hippocampus is the only region of the adult mammalian brain in which there is direct evidence of *de novo* estradiol synthesis (Prange-Kiel et al., 2003), although there is indication of *de novo* estradiol synthesis in the cerebellum of the developing brain as well (Amateau et al., 2004). Interestingly, hippocampal levels of estradiol exceed plasma levels of circulating estradiol (Kato et al., 2013). Together with these previous studies, our findings emphasize the importance of neuroestrogens in hippocampal function after recent loss of ovarian hormones. More extensive examination of the impact of neuroestrogens on other brain regions is warranted.

The results of the current experiments demonstrate for the first time a direct connection between brain-derived estradiol and ERE-dependent transcription *in vivo*. Locally produced estrogens in the brain have been proposed to act as neurotransmitters in a rapid manner (Balthazart et al., 2001). Neuroestrogens influence synaptic plasticity in the hippocampus by initiating nongenomic actions of membrane estrogen receptors, particularly estrogen receptor α (Ishii et al., 2007; Wang et al., 2018). Our results show that inhibition of estradiol synthesis in the brain results in a decrease in ERE-dependent transcription, demonstrating a role for neuroestrogen regulation of the genomic effects of estrogen receptors in addition to the established nongenomic membrane effects. These findings are consistent with the previously suggested hypothesis (Cornil, 2018; Vajaria and Vasudevan, 2018) that the activation of membrane estrogen receptors by neuroestrogens may ultimately lead to transcriptional activity of nuclear estrogen receptors through the activation of various signaling cascades.

In Experiments 3 and 4, we showed that ERE-dependent transcription in the brain decreases after long-term ovariectomy. Estrogen receptor expression in the brain changes after long periods of ovarian hormone deprivation (Bohacek and Daniel, 2009), which likely contributes to this decline in transcriptional activity. Although ERE-dependent transcription significantly decreased in brains of long-term ovariectomized mice, there remained residual activity. Thus, estrogen receptor activity persists even after prolonged ovarian hormone deprivation and likely subsequent changes in estrogen receptor expression in the brain. However, in contrast to the effects seen following short-term ovariectomy, the inhibition of aromatase activity via letrozole did not block residual transcriptional activity after long-term ovariectomy. Therefore, results indicate that remaining transcriptional activity following long-term ovarian hormone deprivation is not mediated by neuroestrogens. To our knowledge, this duration of ovarian hormone deprivation is the longest at which neuroestrogen activity has been tested in rodents. Previous work showed an effect of letrozole on hippocampal spine density in rats at 4 weeks after ovariectomy (Zhou et al., 2010), a time point in between our short-term and long-term paradigms. Future studies should investigate the time course of the decline of neuroestrogen activity following ovariectomy. Nevertheless, these results indicate that there is a point at which neuroestrogens no longer mediate estrogen receptor transcriptional activity following the loss of ovarian function.

This observed decline in neuroestrogen activity following long-term ovariectomy is consistent with previous literature indicating that systemic estrogens are necessary to regulate estradiol synthesis in the brain. Systemic estrogens regulate GnRH release by providing feedback at the level of the hypothalamus (Clarke and Cummins, 1987), which in turn regulates neuroestrogen synthesis in the hippocampus (Prange-Kiel et al., 2013). The administration of GnRH to the hippocampus reverses the loss of spines (Prange-Kiel et al., 2013) and memory impairment (Nelson et al., 2016) associated with aromatase inhibition. A recent study using the newly validated ultra-performance liquid chromatography-tandem mass spectrometry method of measuring estradiol content showed that the brains of female rats have higher levels of estradiol in the hippocampus, amygdala, and preoptic area than in blood serum 24 h after ovariectomy (Li and Gibbs, 2019). Importantly, systemic estradiol treatment increases levels of estradiol in the brain in a dose-dependent manner, but this effect was blocked by systemic letrozole injections. These studies indicate a mechanism through which systemic estrogen regulates neuroestrogen synthesis in the brain in a dose-dependent manner via GnRH signaling. Previous work also demonstrated that letrozole administration following long-term ovariectomy did not result in the same memory impairment shown in the presence of circulating estrogens (Nelson et al., 2016). Collectively, results suggest that prolonged ovarian hormone deprivation would result in a loss of regulation of neuroestrogen synthesis, which could explain why neuroestrogens no longer mediate estrogen receptor activity after long-term ovariectomy.

Because we observed residual ERE-dependent transcription following long-term ovariectomy that was not impacted by letrozole treatment, we can conclude that other mechanisms must be mediating this transcription. Growth factors such as insulin-like growth factor-1 (IGF-1) can influence estrogen receptor-dependent transcription in a ligand-independent manner by activating signaling pathways that lead to phosphorylation of estrogen receptors (Smith, 1998). Estrogen receptors and IGF-1 receptors colocalize in cells in the female rodent brain (Cardona-Gómez et al., 2000), and the administration of IGF-1 to the brains of ovariectomized rats results in increased phosphorylation of estrogen receptor α at serine-118 (Grissom and Daniel, 2016). This ligand-independent mechanism is presumed to be independent not only from ovarian estrogens, but from neuroestrogens as well. However, recent results show that *in vitro* some aromatase activity is necessary for IGF-1 activation of estrogen receptors to occur in the Neuro-2A cell line (Pollard and Daniel, 2019). In the current studies, inhibition of aromatase activity did not impact what is likely ligand-independent activation of estrogen receptors following long-term ovariectomy *in vivo*. These seemingly contradictory observations may be explained by inherent differences in the rapid ligand-independent activation of estrogen receptors *in vitro* and long-term modulation of estrogen receptor function after a prolonged absence of available ligands. Future studies should investigate the interaction between neuroestrogen activity and ligand-independent activation of estrogen receptors *in vivo*.

The results of the current experiments are consistent with previous studies using the ERE-Luciferase reporter mouse model. Earlier studies validating this model demonstrated that luciferase activity is sensitive to estradiol levels in a tissue-specific manner, that the inhibition of estrogen receptors with ICI 182,780 successfully blocks luciferase expression, and that some luciferase activity is still detectable in the brains of ovariectomized adult mice (Ciana et al., 2001, 2003; Stell et al., 2008). Importantly, the current study expands on previous work by implicating neuroestrogens in ERE-dependent transcription in the brain following short-term ovariectomy and by showing that luciferase expression is decreased in the cortex, hypothalamus, and hippocampus following long-term ovariectomy.

Overall, the results of the current experiments indicate that regulation of estrogen receptor dependent transcription in the brain changes following prolonged periods of ovarian hormone deprivation, such as menopause in humans. However, several studies in humans and nonhuman primates suggest that neuroestrogens may still play a role in cognition after menopause. For example, long-term systemic letrozole treatment impaired performance on a hippocampal-dependent memory task in postmenopausal women (Bayer et al., 2015), and in gonadectomized male and female marmosets 4 weeks of systemic letrozole treatment impaired performance on a spatial memory task (Gervais et al., 2019). Interestingly, this latter study also showed an increase in hippocampal estradiol levels in the letrozole-treated marmosets, suggesting a potential rebound effect on neuroestrogen synthesis following long-term letrozole exposure. Whereas these findings may appear inconsistent with the results of the current study—which suggest that the role of neuroestrogens in cognition declines after loss of ovarian function—the inconsistencies may reveal a mechanistic difference in the methods of aromatase inhibition used. Gervais et al. (2019) used a model of long-term systemic letrozole administration based on the regimen frequently used by postmenopausal women with breast cancer, such as those tested in the study by Bayer et al. (2015). Systemic letrozole administration is able to influence the brain by crossing the blood–brain barrier (Dave et al., 2013), but it may also result in changes in peripheral steroid hormone synthesis in adipose or adrenal tissues. On the other hand, the relatively shorter, brain-specific administration of letrozole used in rodent studies, including the present work, is less likely to influence these peripheral tissues. Additionally, a metareview of the literature regarding the effects of endocrine therapies in postmenopausal women revealed mixed findings on the impact of aromatase inhibitors on cognition (Lee et al., 2016), illustrating the complexity of effects of long-term aromatase inhibition on the brain.

The results of the current work point to a continued role for estrogen receptor activation in the brain after loss of ovarian function. Together with other studies, they suggest a potential alternative route for combatting postmenopausal cognitive decline. Previous work has shown that viral–vector-mediated upregulation of estrogen receptor (ER) α in the hippocampi of aging ovariectomized rats enhances memory in the absence of circulating estrogens (Witty et al., 2012). In hippocampal cultures, it has been shown that the administration of an ERα agonist increases spine density, even in the presence of letrozole (Zhou et al., 2010). Other previous work has shown that estrogen receptors in the brain remain transcriptionally active in the absence of ovarian or exogenously delivered estrogens (Pollard et al., 2018). We have now expanded these results to show that estrogen receptors can activate transcription in the brain in the absence of brain-derived estrogens, as well. Overall, these studies represent an increasing body of literature that suggests an important role for estrogen receptors—independent from estrogens—in maintaining cognitive health in the aging female brain.

## Synthesis

Reviewing Editor: Margaret McCarthy, Univ of Maryland School of Medicine Decisions are customarily a result of the Reviewing Editor and the peer reviewers coming together and discussing their recommendations until a consensus is reached. When revisions are invited, a fact-based synthesis statement explaining their decision and outlining what is needed to prepare a revision will be listed below. The following reviewer(s) agreed to reveal their identity: Matthew Paul.

The manuscript advances our understanding of persistent estrogen receptor action in the brain following ovariectomy. It provides evidence that neuroestrogens contribute to this estrogen receptor-dependent transcription in the short term (10 days after ovariectomy), but not long term (70 days after ovariectomy). The studies identify unique time points to measure the possible effects of neuroestrogens and approach possible mechanisms by which the neuroestrogens may be synthesized or may act.

The findings indicate that neuroestrogens contribute to the high level of ERE-transcriptional activity seen after short-term ovariectomy. After long-term ovariectomy, ERE-transcriptional activity was low and neuroestrogens did not appear to contribute to this residual activity. The manuscript is well written, although there is quite a bit of redundancy with respect to the methodology (see comment #1). Statistical methods are appropriate, although the relatively small sample sizes likely hinder some comparisons (see comment #2). The findings are interesting and add to our understanding of the role of neurosteroids in the regulation of estrogen receptor activity.

The main concern of both reviewers is the low power related to analyses of brain regions. The findings in experiment 2 seem to be driven primarily by large differences in the hippocampus with only minor effects in the cortex and hypothalamus. After a discussion, the reviewers agreed that the most desirable outcome is a repeat of Experiment #2 but without the ICI-treatment group. Thus, the study would include the 3 groups; Intact+aCSF, OVX+aCSF and OVX+Letrozole. Increasing the n would allow for clearer conclusions regarding which brain regions are responding to neuroestrogens. The assumption is that this experiment would require less than 2 months work.

The reviewers had additional comments for your consideration.

Specific comments

1. The description of the methods for each experiment is unnecessarily redundant. For example, description of ovariectomy procedures in Experiments 1-4 could be condensed into a single paragraph, with a single sentence specifying the sample sizes within each experiment and another sentence specifying the loss of one ovariectomized mouse in Experiment 3. There is also some redundancy in describing the methods across sections (reiterating the methods in the results section and figure legends), which could be condensed.

2. The authors are correct in pointing out that the effect of Letrozole after short-term ovariectomy is likely driven by the data from the hippocampus. The absence of a significant treatment x brain region interaction is likely due to the relatively small sample size for the number of groups and factors in the analysis and the fact that only the Letrozole treatment differs across brain regions. In order to detect the treatment x brain region interaction, a larger sample size or more focused analysis is likely needed. The authors should include a discussion of these statistical issues in the Discussion section.

3. The significance statement focuses on estrogen actions on cognitive function, but the study does not measure cognitive function in the present experiment. I suggest focusing the significance statement on the regulation of estrogen receptor transcriptional activity, with perhaps a sentence or two referring to the potential behavioral ramifications of this regulation (which includes, but is not limited to cognitive function).

4. At what time of day were mice sacrificed? Were all mice within each experiment sacrificed at the same time of day? Are there any studies that have determined whether ERE transcriptional activation fluctuates across the day/night cycle?

5. Is there a way to determine or correct for background luminance in the RLU measures? Can the authors rule out the possibility the residual RLU after long-term ovariectomy is not simply background luminance?

Minor comments

6. Abstract, line 15. I recommend specifying that the effect of ICI occurred in 10-day ovariectomized mice to remove any ambiguity.

7. Line 168, “Three days following stereotaxic surgery, ...” This phrase only applies to Experiments 2 and 4. Given that the timelines are covered in a figure as well as other sections in the methods, this clause can be replaced with, “At sacrifice, ...”

8. Lines 167-191. The authors should cite previous studies that have used/validated the luciferase quantification procedures in the Methods section.

9. I presume the sample sizes listed in the statistics tables are the final sample sizes after attrition due to surgical death and tissue damage during extraction. It would help the reader if the authors noted in the methods that the final sample sizes are listed in these tables.

10. Figure 2B. Symbols should not be used for near significant results. Instead, the p value can be directly stated in place of the symbol.

11. Line 229. According to the methods and Figure 1B, cannula and minipump implantation was conducted 7 days after ovariectomy or sham surgery, not 10 days.

12. It would help the reader if significant findings were indicated on the graphs of Figures 3-5, rather than just in the figure legends.

## Author Response

Response to Reviewers

We thank the reviewers for their thoughtful consideration of our paper. Our responses are written below each comment in italics. In the revised manuscript, our changes are written in red font.

The findings indicate that neuroestrogens contribute to the high level of ERE-transcriptional activity seen after short-term ovariectomy. After long-term ovariectomy, ERE-transcriptional activity was low and neuroestrogens did not appear to contribute to this residual activity. The manuscript is well written, although there is quite a bit of redundancy with respect to the methodology (see comment #1). Statistical methods are appropriate, although the relatively small sample sizes likely hinder some comparisons (see comment #2). The findings are interesting and add to our understanding of the role of neurosteroids in the regulation of estrogen receptor activity.

Please see our responses to the issues mentioned above under Specific Comments #1 and #2.

The main concern of both reviewers is the low power related to analyses of brain regions. The findings in experiment 2 seem to be driven primarily by large differences in the hippocampus with only minor effects in the cortex and hypothalamus. After a discussion, the reviewers agreed that the most desirable outcome is a repeat of Experiment #2 but without the ICI-treatment group. Thus, the study would include the 3 groups; Intact+aCSF, OVX+aCSF and OVX+Letrozole. Increasing the n would allow for clearer conclusions regarding which brain regions are responding to neuroestrogens. The assumption is that this experiment would require less than 2 months work.

We appreciate the reviewers' suggestion to repeat Experiment 2 in order to more accurately be able to draw brain region-specific conclusions regarding neuroestrogen activity. Although we think the request is reasonable, we do have practical issues that will impede our ability to repeat the experiment in the reviewers' assumed time frame of two months. Our ERE-Luc colony is currently small. We currently have only 10 heterozygous mice “ready to go”. However, eight are 9-months of age (planned for an aging study) and only two are young adults (70 days of age - similar in age to those used our experiments). We are concerned that combining results from the young adult and middle-aged animals is problematic and do not consider this option viable. With the number of breeding pairs we currently have, we predict it would take us ~6 months to breed enough animals to complete the experiment as requested. We therefore chose to more thoroughly discuss the limitations of the data in the text. We feel that the main conclusions drawn are justifiable based on the data and that we have reasonably addressed the concerns expressed by the reviewers in the Discussion. Please see our response to Specific Comment #2 to note the changes we made to the text.

The reviewers had additional comments for your consideration.

Specific comments

1. The description of the methods for each experiment is unnecessarily redundant. For example, description of ovariectomy procedures in Experiments 1-4 could be condensed into a single paragraph, with a single sentence specifying the sample sizes within each experiment and another sentence specifying the loss of one ovariectomized mouse in Experiment 3. There is also some redundancy in describing the methods across sections (reiterating the methods in the results section and figure legends), which could be condensed.

We appreciate this comment and have made extensive revisions to reduce redundancy of the methods descriptions throughout the paper. As suggested, we have condensed ovariectomy procedures section and have also removed some redundancy in the estrous cycle tracking and cannula mini-pump implantations sections of the methods (See pages 6-8). Additionally, we have removed descriptions of the methods from the results section (pages 11-13). However, we decided to leave short descriptions of the methods in the figure legends, based on the instructions from eNeuro's Guidelines for Authors that states that figure legends should provide the reader with sufficient detail to understand the figure without referencing the text. We did remove non-significant p values to better streamline the figure legends.

2. The authors are correct in pointing out that the effect of Letrozole after short-term ovariectomy is likely driven by the data from the hippocampus. The absence of a significant treatment x brain region interaction is likely due to the relatively small sample size for the number of groups and factors in the analysis and the fact that only the Letrozole treatment differs across brain regions. In order to detect the treatment x brain region interaction, a larger sample size or more focused analysis is likely needed. The authors should include a discussion of these statistical issues in the Discussion section.

We agree that the absence of a significant brain region x treatment interaction may be due to the relatively small sample size, but we believe our statistical methods are the most appropriate for the questions of our study. Due to the practical limitations described above, we chose to de-emphasize the brain region specific effects and add more to the discussion section addressing these problems. We removed a statement from the results section of the manuscript that suggested the main effect of letrozole treatment was driven by the large decrease in the hippocampus (“an effect that appears to be primarily driven by the effect on the hippocampus (see Figure 3)”, under the Experiment 2 part of the Results section). We added several lines to the Discussion section addressing the limitations of our conclusions due to sample size and statistics (page 14). Additionally, we reframed the paragraph in the Discussion that focuses on the hippocampus to acknowledge that the hippocampus is where the bulk of the literature on neuroestrogens in mammals has focused on, rather than emphasizing our findings in the hippocampus (page 14).

3. The significance statement focuses on estrogen actions on cognitive function, but the study does not measure cognitive function in the present experiment. I suggest focusing the significance statement on the regulation of estrogen receptor transcriptional activity, with perhaps a sentence or two referring to the potential behavioral ramifications of this regulation (which includes, but is not limited to cognitive function).

We agree that the significance statement should focus on estrogen receptor function rather than cognition. We have edited the statement to reflect that (page 3).

4. At what time of day were mice sacrificed? Were all mice within each experiment sacrificed at the same time of day? Are there any studies that have determined whether ERE transcriptional activation fluctuates across the day/night cycle?

We do not believe that daily fluctuation of ERE transcriptional activity has been shown using this mouse model. However, animals in all experiments were killed within the same time window (10:00am-12:00pm) in order to coincide with the timing of the daily vaginal smears. We have added a sentence to the methods section (line #159-160) to include this information in the manuscript.

5. Is there a way to determine or correct for background luminance in the RLU measures? Can the authors rule out the possibility the residual RLU after long-term ovariectomy is not simply background luminance?

In order to check for background luminescence in the RLU measures of the samples, we take baseline readings of the samples before luciferin is added. These baseline measurements are negligible, and orders of magnitude smaller than the readings after luciferin is added (Baseline readings are consistently ~25 RLUs whereas readings of activated samples are consistently in the hundreds-thousands range). Additionally, we believe that the residual activity in the long term samples is not simply background luminescence because it is possible to achieve lower levels of relative luminescence with the administration of ICI, such as was shown in Experiment 2.

Minor comments

6. Abstract, line 15. I recommend specifying that the effect of ICI occurred in 10-day ovariectomized mice to remove any ambiguity.

We appreciate this comment and have edited as suggested for clarification.

7. Line 168, “Three days following stereotaxic surgery, ...” This phrase only applies to Experiments 2 and 4. Given that the timelines are covered in a figure as well as other sections in the methods, this clause can be replaced with, “At sacrifice, ...”

We thank you for this suggestion. Because the heading for this section contains the word “euthanasia”, we decided to remove the beginning clause of the sentence entirely (line #158).

8. Lines 167-191. The authors should cite previous studies that have used/validated the luciferase quantification procedures in the Methods section.

We have added citations for previous studies using this method in line # 183.

9. I presume the sample sizes listed in the statistics tables are the final sample sizes after attrition due to surgical death and tissue damage during extraction. It would help the reader if the authors noted in the methods that the final sample sizes are listed in these tables.

We agree that this statement would help the reader and have added it in line #166.

10. Figure 2B. Symbols should not be used for near significant results. Instead, the p value can be directly stated in place of the symbol.

We have edited Figure 2B to include the p value rather than a symbol.

11. Line 229. According to the methods and Figure 1B, cannula and minipump implantation was conducted 7 days after ovariectomy or sham surgery, not 10 days.

We appreciate the reviewer catching this error. In order to reduce redundancy (Specific comment #1), we have removed the paragraph that contained this sentence.

12. It would help the reader if significant findings were indicated on the graphs of Figures 3-5, rather than just in the figure legends.

We have added asterisks to the group names in Figures 3-5 in order to indicate group differences in comparison to the OVX control groups.
